# Translating Basic Science to Clinical Applications: A Narrative Review of Repurposed Pharmacological Agents in Preclinical Models of Diabetic Neuropathy

**DOI:** 10.3390/biomedicines13071709

**Published:** 2025-07-13

**Authors:** Corina Andrei, Oana Cristina Șeremet, Ciprian Pușcașu, Anca Zanfirescu

**Affiliations:** Faculty of Pharmacy, “Carol Davila” University of Medicine and Pharmacy, Traian Vuia 6, 020956 Bucharest, Romania; corina.andrei@umfcd.ro (C.A.); oana.seremet@umfcd.ro (O.C.Ș.); anca.zanfirescu@umfcd.ro (A.Z.)

**Keywords:** diabetic neuropathy, preclinical trials, neuropathic pain, drug repurposing

## Abstract

Diabetic neuropathy (DN) remains a major clinical burden, characterized by progressive sensory dysfunction, pain, and impaired quality of life. Despite the available symptomatic treatments, there is a pressing need for disease-modifying therapies. In recent years, preclinical research has highlighted the potential of repurposed pharmacological agents, originally developed for other indications, to target key mechanisms of DN. This narrative review examines the main pathophysiological pathways involved in DN, including metabolic imbalance, oxidative stress, neuroinflammation, ion channel dysfunction, and mitochondrial impairment. A wide array of repurposed drugs—including antidiabetics (metformin, empagliflozin, gliclazide, semaglutide, and pioglitazone), antihypertensives (amlodipine, telmisartan, aliskiren, and rilmenidine), lipid-lowering agents (atorvastatin and alirocumab), anticonvulsants (topiramate and retigabine), antioxidant and neuroprotective agents (melatonin), and muscarinic receptor antagonists (pirenzepine, oxybutynin, and atropine)—have shown promising results in rodent models, reducing neuropathic pain behaviors and modulating underlying disease mechanisms. By bridging basic mechanistic insights with pharmacological interventions, this review aims to support translational progress toward mechanism-based therapies for DN.

## 1. Introduction

Diabetic neuropathy (DN) is one of the most prevalent and debilitating complications of diabetes mellitus, affecting up to 50% of individuals with type 1 or type 2 diabetes. Among its various forms, diabetic peripheral neuropathy is the most common, characterized by progressive, asymmetrical, length-dependent nerve damage that typically begins distally in the lower limbs. Other subtypes include autonomic neuropathy, proximal neuropathy (diabetic amyotrophy), and focal or multifocal neuropathies, each presenting with distinct clinical features [1].

Characterized by progressive, symmetrical nerve damage, DN often presents with a combination of sensory loss and neuropathic pain, such as burning, tingling, or electric shock-like sensations, typically starting in the distal lower extremities [2]. Over time, these symptoms can severely impair the quality of life, increase the risk of foot ulcers, and lead to lower limb amputations [3]. The likelihood of developing DN rises with longer disease duration, inadequate glycemic control, and additional cardiovascular risk factors [4].

Despite advances in understanding DN pathogenesis, current treatment options remain largely symptomatic and insufficient. Pain relief is the primary treatment goal, as it can improve physical function, sleep, mood, and overall well-being, enhancing the quality of life and promoting physical activity [5]. First-line pharmacological therapies—including tricyclic antidepressants, serotonin–norepinephrine reuptake inhibitors, and gabapentinoids [6]—are often limited by modest efficacy and undesirable side effects [7,8,9,10,11,12,13,14]. Moreover, only a minority of patients achieve significant pain relief, underscoring the urgent need for more effective disease-modifying interventions [15].

Growing preclinical evidence highlights the potential of drug repurposing—using existing pharmacological agents developed for other indications—to target the underlying mechanisms of DN. Compounds originally approved for metabolic disorders, neurodegenerative diseases, or cardiovascular conditions have demonstrated promising effects in animal models of DN, modulating key pathogenic processes, such as oxidative stress, neuroinflammation, ion channel dysfunction, and mitochondrial impairment [16,17,18].

However, despite this emerging body of research, there remains a lack of comprehensive, mechanism-focused reviews that integrate these preclinical findings with the broader pathophysiological context of DN.

This narrative review addresses this gap by providing a structured overview of repurposed pharmacological agents with demonstrated efficacy in preclinical models of DN. We categorize these agents based on their primary mechanisms of action, link them to specific pathophysiological targets, and critically evaluate their potential for clinical application. By bridging fundamental research with therapeutic development, this review aims to support the translation of basic science into effective, mechanism-based treatments for diabetic neuropathy.

## 2. Materials and Methods

A focused literature review was conducted using PubMed to identify relevant in vivo preclinical studies investigating potential therapeutic agents for DN. The search was limited to English-language publications, prioritizing recent studies from 2020 to 2025 and considering key older research. The following keywords and MeSH terms were used: “preclinical”, “rat”, “mice”, OR “rodents” AND “diabetic neuropathic pain”, “diabetic neuropathy”, OR “peripheral neuropathy”. Inclusion criteria were designed to ensure study quality and relevance. Eligible studies had to (1) be original research articles published in peer-reviewed journals; (2) involve rodent models of DN; and (3) report significant findings on therapeutic agents, whether used alone or in combination. Additionally, studies needed to provide details on drug names, dosages, mechanisms of action, and the species used. Studies not including this information were excluded.

## 3. Pathogenesis of DN

**Figure 1:**
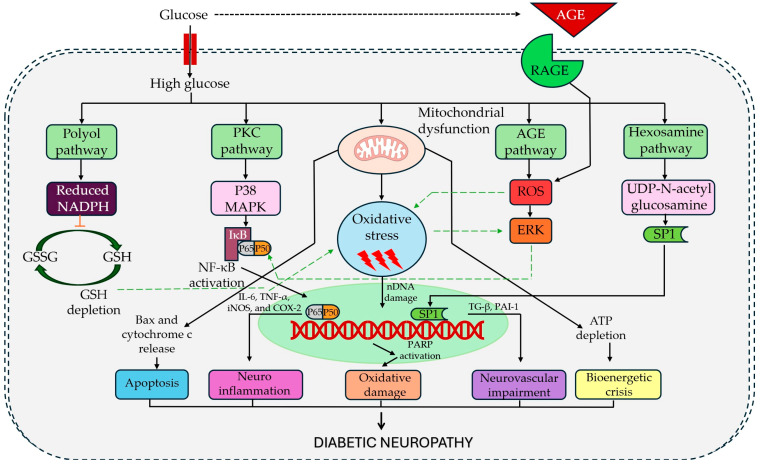
Pathophysiological basis of diabetic neuropathy. Legend: AGE, advanced glycation end-products; RAGE, receptor for advanced glycation end-products; ROS, reactive oxygen species; ERK, extracellular signal-regulated kinase; PKC, protein kinase C; P38 MAPK, p38 mitogen-activated protein kinase; NF-κB, nuclear factor kappa-light-chain-enhancer of activated B cells; IκB, inhibitor of NF-κB; IL-6, interleukin-6; TNF-α, tumor necrosis factor-alpha; iNOS, inducible nitric oxide synthase; COX-2, cyclooxygenase-2; GSH, reduced glutathione; GSSG, oxidized glutathione; NADPH, nicotinamide adenine dinucleotide phosphate (reduced form); Bax, Bcl-2-associated X protein; SPI, specificity protein 1; nDNA, nuclear DNA; PARP, poly (ADP-ribose) polymerase; TGF-β, transforming growth factor-beta; PAI-1, plasminogen activator inhibitor-1; SPT1, serine palmitoyltransferase long chain base subunit 1; UDP-N-acetylglucosamine, uridine diphosphate N-acetylglucosamine; ATP, adenosine triphosphate.

DN is a complex, multifactorial disorder driven by a convergence of metabolic abnormalities and neuroimmune dysfunction. Chronic hyperglycemia, often accompanied by dyslipidemia and insulin resistance, triggers a cascade of injurious pathways that ultimately damage peripheral nerves [19]. These include the hyperactivity of glucose metabolic routes (polyol, protein kinase C, hexosamine, and advanced glycation end-product pathways) that generate toxic intermediates and oxidative stress, impairing the microvasculature that nourishes the nerves. The result is metabolic dysfunction and microvascular damage leading to nerve fiber degeneration, Schwann cell injury, and demyelination [19,20]. In parallel, diabetes induces oxidative stress and neuroinflammation, contributing to neural injury and pain sensitization [19,20]. Importantly, these molecular insults also disrupt neuronal ion channel function, promoting ion channel modulation and neuronal hyperexcitability in primary afferents and setting the stage for central sensitization within the spinal cord [20]. Together, these mechanisms form the pathophysiological basis of DN and highlight numerous potential targets for therapeutic intervention (Figure 1).

### 3.1. Metabolic Dysfunction and Microvascular Damage

Persistent hyperglycemia disrupts normal metabolism, leading to toxic metabolite buildup that damages peripheral nerves. Excess glucose is channeled into the polyol, hexosamine, and protein kinase C (PKC) pathways that contribute to the generation of advanced glycation end-products (AGEs). Each pathway contributes to neuronal injury. Sorbitol depletes myoinositol and impairs Na^+^/K^+^-ATPase, affecting axonal conduction [21]. AGEs alter proteins and activate specific receptors, inducing oxidative stress and inflammation [21].

At the same time, insulin resistance or deficiency reduces neurotrophic support, promoting axonal atrophy and impairing repair [22]. Diabetes-induced dyslipidemia causes lipid buildup in nerves, increasing oxidative damage and inflammation [22].

Beyond metabolic toxicity, diabetes disrupts the microvascular supply to peripheral nerves. Chronic hyperglycemia impairs the vasa nervorum, leading to basement membrane thickening and endothelial dysfunction [23,24,25,26]. This process is exacerbated by PKC activation, which alters the expression of the vascular endothelial growth factor and promotes vasoconstriction [27,28]. The resulting reduction in perfusion causes endoneurial hypoxia and energy failure, contributing to axonal degeneration and demyelination [29,30]. In painful DN, these microvascular abnormalities are even more pronounced, with altered skin microvascular regulation [31]. These findings underscore how metabolic and vascular dysfunction converge to drive nerve injury in DN.

### 3.2. Oxidative Stress

Oxidative stress is a key driver of DN, fueled by an excessive production of reactive species of oxygen (ROS) and impaired antioxidant defenses [32]. Hyperglycemia promotes ROS generation through mitochondrial overload, NADPH oxidase 2/4 activation [33,34], and glucose or lipid auto-oxidation [35,36]. The reactive species damage neurons and Schwann cells by oxidizing DNA, lipids, and proteins [37,38]. Although Schwann cells initially mount antioxidant responses, prolonged ROS exposure causes mitochondrial dysfunction, NADPH oxidase 4-mediated stress, and apoptosis [34,39,40]. ROS also activate PKC and c-Jun N-terminal kinase, further amplifying nerve injury [41,42].

Lipids also contribute to nerve damage [1]. Diabetic nerves exhibit increased lipid peroxidation and DNA oxidation, correlating with neuropathy severity [43]. Cholesterol-derived oxysterols, along with oxidized LDL, activate inflammatory signaling via LOX-1, Toll-like receptor 4, and AGE receptors [37,44,45,46,47]. This cascade promotes caspase-3 activation, nuclear DNA degradation, and neuronal damage, promoting inflammation, oxidative stress, and nerve damage [37,44,45,46,47].

Importantly, oxidative stress is not only a consequence but also a driver of metabolic imbalance. ROS activate poly(ADP-ribose) polymerase, depleting NAD^+^, impairing glycolysis, and enhancing polyol and AGE pathway activity, creating a vicious cycle that accelerates nerve fiber loss.

### 3.3. Neuroinflammation

Once considered a purely metabolic complication, DN is now recognized as a disorder with a strong neuroinflammatory component. Chronic hyperglycemia induces low-grade systemic inflammation, reflected by elevated proinflammatory cytokines and acute-phase reactants, which correlate directly with the onset and progression of diabetic polyneuropathy [48]. In the peripheral nerves, hyperglycemia and dyslipidemia disrupt endothelial integrity, increasing blood–nerve barrier permeability and allowing monocyte and T-cell infiltration [49]. Activated macrophages and lymphocytes release cytokines and chemokines that damage neurons and Schwann cells while sensitizing nociceptors. Experimental models show that endoneurial macrophage buildup is associated with small fiber loss and NeP [43]. Additionally, hyperglycemia impairs Schwann cell function, reducing neurotrophic factor levels while increasing proinflammatory signaling, including CXCR3-mediated T-cell recruitment [50].

Neuroinflammation extends beyond peripheral nerves, with immune activation in the central nervous system playing a key role in NeP. Diabetes and nerve injury activate dorsal horn microglia that become hypertrophic and express ionized calcium-binding adaptor molecule 1 [51,52]. Hyperglycemia and ATP further stimulate P2X receptors, promoting cytokine release and amplifying nociceptive signaling [53]. Microglia-derived brain-derived neurotrophic factor disrupts chloride homeostasis, reducing inhibitory control in dorsal horn neurons [54], while astrocyte activation reinforces central sensitization through chemokines and glutamate release.

### 3.4. Ion Channel Modulation and Neuronal Hyperexcitability

A hallmark of diabetic NeP is sensory neuron hyperexcitability. Despite the axonal loss, surviving nociceptors develop spontaneous activity due to diabetes-induced changes in ion channel expression and function [22,55].

Several voltage-gated sodium channels, including Nav1.3, Nav1.6, Nav1.7, and Nav1.9, were upregulated in the dorsal root ganglia of streptozotocin-induced diabetic rodents. These changes contribute to the development of thermal and mechanical allodynia [56]. Furthermore, gain-of-function mutations in the *SCN9A* gene, which encodes Nav1.7, have been directly linked to painful DN in humans [57].

Hyperglycemia also elevates methylglyoxal, a glycolytic byproduct that modifies Nav1.8 and TRPA1 channels, further increasing nociceptor sensitivity [58,59,60,61]. Similarly, the heat-sensitive capsaicin receptors TRPV1 are also upregulated or sensitized in DN, possibly via PKC-mediated phosphorylation, contributing to burning pain [62].

Other excitatory channels, including T-type calcium channels such as Cav3.2, N-methyl-D-aspartate (NMDA), and P2X receptors, are frequently upregulated, amplifying nociceptive transmission [63,64,65]. Meanwhile, inhibitory mechanisms are diminished; potassium channels, such as Kv1, Kv7, and ATP-sensitive potassium channels, are down-regulated in diabetic rodents, disrupting neuronal repolarization, promoting ectopic firing, and facilitating central sensitization [66].

### 3.5. Mitochondrial Dysfunction

Mitochondrial dysfunction and impaired energy metabolism are key features of hyperglycemia-induced nerve damage [67,68]. Chronic hyperglycemia alters mitochondrial membrane potential, impairing respiration and ATP production [69,70,71].

This dysfunction contributes to axonal degeneration, particularly through toxic acylcarnitine accumulation in Schwann cells, dorsal root ganglion neurons, and axons [72]. Additionally, elevated free fatty acids, undergoing excessive β-oxidation due to hyperlipidemia, further damage peripheral nerves—especially Schwann cells [73]—by increasing ROS production and promoting inflammation. This inflammatory response is amplified by activated macrophages, which release cytokines and chemokines, perpetuating nerve injury [74].

### 3.6. Central Sensitization

In diabetes, persistent nociceptor activity and neuroinflammation drive central sensitization—an exaggerated pain response within the central nervous system. In DN, ongoing peripheral input increases excitatory neurotransmitters, such as glutamate and substance P, in the dorsal horn, amplifying pain signaling. NMDA receptor overactivation and reduced inhibitory control contribute to allodynia. Additionally, activated microglia and astrocytes release proinflammatory cytokines and brain-derived neurotrophic factor that reduce GABA-mediated inhibition. These cytokines further enhance NMDA receptor activity and activate intracellular kinases, perpetuating a sensitized state [75].

In animal models of diabetes, increased extracellular signal-regulated kinase (ERK) phosphorylation in spinal neurons and astrocytes correlates with mechanical hypersensitivity, while ERK inhibition reduces pain behavior. These central changes can become self-sustaining, allowing pain to persist even when glucose levels normalize [75].

Painful DN also involves supraspinal changes. Neuroimaging studies reveal cortical reorganization, with reduced gray matter in the somatosensory cortex and increased activity in pain-related regions such as the insula and anterior cingulate cortex [76]. Altered connectivity is also seen in ascending and descending pathways [77,78,79]. Although it remains unclear whether these changes are a cause or consequence of chronic pain, they reinforce the role of brain-level central sensitization in DN.

Oxidative stress, inflammation, and ion channel dysfunction are interrelated mechanisms that progressively amplify each other in painful DN. Hyperglycemia-driven oxidative stress activates redox-sensitive pathways like the nuclear factor kappa-light-chain-enhancer of activated B cells (NF-κB) pathway [80], leading to proinflammatory cytokine release, which amplifies oxidative injury and disrupts Schwann cell function [81,82]. These inflammatory mediators also alter ion channel expression and activity, enhancing nociceptor excitability [83,84]. Aberrant neuronal firing promotes glial and immune activation, creating a vicious cycle of inflammation, oxidative stress, and neurodegeneration that drives chronic pain and demyelination in DN [85].

## 4. Substances with Potential Effectiveness in Treating DN

Various pharmacological substances have been investigated in animal models of DN, targeting both symptom management and underlying disease mechanisms. These compounds exhibit diverse modes of action, including anti-inflammatory, antioxidant, neuroprotective, and ion channel-modulating effects. Some primarily attenuate NeP—symptoms such as mechanical allodynia and thermal hyperalgesia—while others show potential for slowing or reversing neurodegenerative processes.

Most preclinical studies rely on genetic or chemically induced models, using streptozotocin or alloxan, which cause selective destruction of pancreatic β-cells and persistent hyperglycemia. These models are widely accepted for replicating key features of type 1 diabetes and its complications, including sensory hypersensitivity, oxidative stress, nerve fiber loss, and neuroinflammation.

As discussed in Section 3, mitochondrial dysfunction, oxidative stress, and chronic low-grade inflammation are key contributors to peripheral nerve damage in DN. Consequently, pharmacological agents that restore metabolic balance, improve mitochondrial function, and exert antioxidant and anti-inflammatory effects are of growing interest as potential disease-modifying therapies. Table 1 summarizes preclinical studies investigating such compounds in experimental models of DN. These agents, which have already been approved for metabolic or cardiovascular indications, have shown the ability to reduce oxidative stress, modulate inflammatory pathways, and improve mitochondrial homeostasis, ultimately alleviating neuropathic symptoms.

Mitochondrial dysfunction and oxidative stress are central drivers of neuronal injury in DN. However, experimental evidence increasingly shows that agents targeting these pathways rarely exert isolated effects. Instead, antioxidant and mitochondrial protective compounds often engage overlapping mechanisms that include anti-inflammatory actions and direct neuroprotection, collectively contributing to improved nerve function and pain relief.

Table 2 summarizes pharmacological agents evaluated in animal models of DN that demonstrate this multi-dimensional activity profile. These substances, originally developed for diverse clinical indications, show the ability to attenuate oxidative damage, preserve mitochondrial function, modulate inflammatory responses, and support structural and functional nerve integrity. This mechanistic overlap highlights the interconnected nature of oxidative stress, mitochondrial failure, inflammation, and neurodegeneration in DN pathogenesis.

Increased proinflammatory cytokine signaling, microglial activation, and dysfunction in pain modulatory circuits contribute to both peripheral nerve injury and central sensitization, sustaining chronic pain even when metabolic factors are addressed. A growing number of pharmacological agents have demonstrated the ability to modulate these pathways in experimental models of DN by attenuating cytokine production, suppressing glial activation, or restoring neuroprotective signaling. Many of these compounds act at the intersection of inflammation, immune activation, and pain processing, offering promising therapeutic potential. Table 3 summarizes these agents, detailing their effects on neuroinflammatory mediators, central sensitization markers, and pain-related outcomes in animal models of DN.

Aberrant neuronal excitability is a key contributor to neuropathic pain and sensory deficits in diabetic neuropathy (DN). Targeting ion channels and pathways involved in neuronal hyperactivity has emerged as a promising strategy for symptom relief and potential disease modification. Table 4 summarizes preclinical studies evaluating repurposed pharmacological agents that modulate ion channels or regulate neuronal excitability in animal models of DN.

## 5. Discussion

This review highlights emerging evidence for repurposed pharmacological agents in DN, focusing on their analgesic efficacy, disease-modifying effects, and underlying mechanisms based on preclinical studies. By integrating findings from animal models, we explore how these compounds influence pain signaling, neuroinflammation, oxidative stress, and neurodegeneration.

### 5.1. Modulating Neuronal Hyperexcitability

Retigabine [140], reboxetine [126], and ifenprodil [127] significantly reduced mechanical and thermal hypersensitivity in animal models by modulating abnormal neuronal firing and enhancing descending inhibitory pathways [126,127,140]. Cilostazol improved mechanical allodynia and preserved nociceptive fibers without affecting thermal pain, suggesting selective modulation of Aβ and Aδ fibers [142].

Additional agents such as drofenine [151] and N-acetylcysteine [144] further support the role of ion channel modulation in DN, improving sensory response and reducing pain [151] via TRPV1, NMDA, and P2X7 pathways [144]. Muscarinic M1 receptor antagonists (pirenzepine, atropine, oxybutynin) further demonstrated robust analgesic and neuroprotective effects [146,148].

### 5.2. Mitigating Inflammation and Oxidative Stress

Chronic inflammation and oxidative stress are central to both pain and neurodegeneration in DN. Agents such as telmisartan [123], clavulanic acid [124], metformin [88,135], melatonin [114,156], pentoxifylline [120,133], semaglutide [90], and atorvastatin [91] consistently reduced inflammatory cytokines (TNF-α, IL-1β, IL-6) and oxidative markers (MDA, ROS), correlating with improved behavioral and structural outcomes.

Etifoxine [129] and bupivacaine [131] also reduced inflammation and improved pain thresholds. Topiramate enhanced neuroprotection via glial modulation [18], while liraglutide targeted central inflammation through NLRP3 inhibition [102].

### 5.3. Promoting Neuroprotection, Remyelination, and Mitochondrial Function

Alirocumab [93], pramipexole [122], melatonin [156], and tropisetron [115] promoted nerve preservation by enhancing neurotrophic factors and mitochondrial biogenesis. Vincamine [112] and amlodipine [106] improved mitochondrial function via AMPK/SIRT1/PGC-1α signaling, which is crucial for maintaining neuronal energy homeostasis. Romidepsin, a histone deacetylase inhibitor, facilitated nerve regeneration through epigenetic modulation [118].

### 5.4. Modulating Glial Activation and Central Sensitization

Central sensitization and glial activation are major drivers of chronic pain. Clavulanic acid [124], ifenprodil [127], semaglutide [90], cilostazol [142], etifoxine [129], topiramate [18], and bupivacaine [131] reduced microglial reactivity and inflammatory cytokine release, indicating that glia-targeted therapies may stabilize pain processing circuits.

### 5.5. Mitigating Advanced Glycation and Metabolic Dysregulation

Gliclazide [98] lowered sorbitol buildup and oxidative stress, while alirocumab [93] and atorvastatin [91] improved lipid profiles and suppressed AGE signaling.

Topiroxostat, a xanthine oxidase inhibitor, preserved nerve function and structure by inhibiting macrophage polarization and reducing systemic oxidative stress, highlighting its dual antioxidant and anti-inflammatory capacity [104].

Metformin alone or associated with duloxetine, oxycodone, or vitamin B12 consistently showed enhanced efficacy in reducing pain and improving neural integrity, driven by metabolic regulation via AMPK activation and ROS reduction [95].

### 5.6. Multi-Targeted Approaches

Combination therapies offer synergistic effects across several pathogenic pathways. Melatonin + gabapentin [156], rolipram + pentoxifylline [133], and sildenafil + metformin [135,137] demonstrated enhanced antinociceptive and neuroprotective outcomes.

Similarly, the combination of agomelatine + morphine yielded longer-lasting analgesia while attenuating morphine tolerance, suggesting that melatonergic signaling could be harnessed to optimize opioid therapy [153].

Metformin co-administered with various agents or administered intrathecally displayed dose-sparing effects, broadening its translational value [95,135,137].

**Figure 2:**
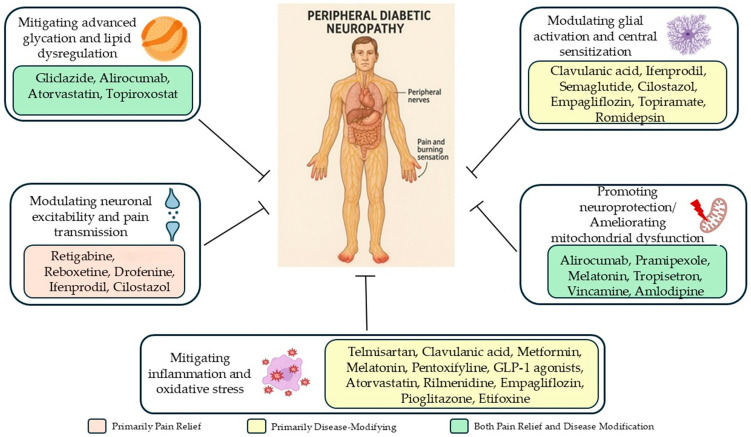
Pathophysiological mechanisms targeted by repurposed drugs in diabetic neuropathy: from pain relief to disease modification.

Figure 2 summarizes the mechanisms of action of these repurposed antineuropathic agents.

Although current clinical guidelines for the management of DN primarily recommend agents such as duloxetine, pregabalin, and tricyclic antidepressants for symptomatic relief, these drugs are often limited by partial efficacy and side effects. The repurposed pharmacological agents discussed here, while not currently included in guideline-based treatment algorithms, may complement or enhance existing therapies through disease-modifying effects. Their ability to target multiple underlying mechanisms—such as neuroinflammation, oxidative stress, and mitochondrial dysfunction—offers a promising direction for expanding the therapeutic landscape.

However, clinical validation is necessary before these compounds can be integrated into evidence-based treatment strategies. Despite promising preclinical results, clinical translation remains inconsistent and challenging.

Several compounds that exhibited therapeutic efficacy in rodent models did not translate into clinical success. Liraglutide (1.2–1.8 mg/day subcutaneously for 26 weeks) reduced inflammation but failed to improve neuropathy outcomes in humans, possibly due to the advanced stage of neuropathic damage in the tested patients [158]. Similarly, pentoxifylline was unable to demonstrate clinical efficacy in a one-year randomized controlled trial [159], while the utility of topiramate has been limited by adverse effects, including sensory disturbances, fatigue, and cognitive dysfunction [160].

Other agents have produced varied outcomes. Telmisartan (40–80 mg) [161] and aliskiren [162] reduced symptoms and inflammation in small trials, while cilostazol (100–200 mg) improved walking speed but not neuropathic symptoms [163]. Clinical data on metformin remain conflicting, with some studies reporting nerve preservation [164] and others indicating increased DN risk at high doses, possibly linked to metformin-induced vitamin B12 deficiency [165].

Encouragingly, empagliflozin (25 mg/day) reduced pain scores, improved electrophysiology, and decreased serum levels of neuron-specific enolase and MDA [166]. Semaglutide, combined with cagrilintide, is under investigation in painful DN [167].

The variability in animal models, disease stage, dosing regimens, and outcome measures limits direct clinical applicability. Many repurposed agents show efficacy in early-stage DN under controlled laboratory conditions. Furthermore, species-specific differences in drug metabolism and pain processing can obscure clinical relevance. The lack of standardized biomarkers for early detection and treatment response in DN complicates trial design and therapeutic validation.

To advance these preclinical discoveries toward clinical application, future research should prioritize rigorous, standardized animal models that better mimic human DN progression—including type 2 diabetes and mixed neuropathies. Longitudinal studies examining pain relief and neuroprotection are essential to assess true disease-modifying potential. Combining pharmacological agents targeting distinct mechanisms (e.g., inflammation, oxidative stress, and ion channel modulation) may enhance efficacy and reduce required doses.

## 6. Conclusions

Repurposed pharmacological agents show potential for improving DN management by targeting key pathogenic mechanisms. Greater clinical benefits are likely achieved through multi-targeted therapies that address both symptom control and disease progression. However, rigorous clinical validation is essential before these approaches can be integrated into evidence-based care.

## Figures and Tables

**Table 1:** Metabolic and mitochondrial modulators with antioxidant and anti-inflammatory effects in animal models of DN.

Tested Substance	Approved Indication	Mode of Administration	Animal Strain	Results	Molecular Mechanism	Reference
Metformin	Type 2 diabetes mellitus	200 mg/kg i.p.	Sprague–Dawley rats	Attenuated mechanical allodynia.	Down-regulation of NF-κB. Activation of AMPK signaling pathways in dorsal root ganglion.	[16]
		30, 200, and 500 mg/kg i.p.	Sprague–Dawley rats	Attenuated mechanical and heat hyperalgesia and cold allodynia.	⇑ SOD ⇑ AMPK target genes in sciatic nerves ⇓ MDA ⇓ glycation end-products	[86]
		400 mg/kg orally	Sprague–Dawley rats	Ameliorated tactile hypersensitivity.	⇑ expression of miRNA-146a in RSCs. ⇑ the number and structure of myelinated fibers in the SC. ⇑ SOD ⇑ MNCV ⇑ SNCV ⇓ TNF-α ⇓ p65 ⇓ NO	[87]
		150 mg/kg i.p.	MKR mice	Reduced thermal hypoalgesia, as shown by a significantly lower hind paw withdrawal latency, indicating restored thermal pain perception. Enhanced neuromuscular strength, evidenced by an increased grip strength and longer latency to fall. Exhibited better sensorimotor coordination and performance.	Activation of the AMPK signaling pathway. Restoration of neuronal integrity. ⇓ ROS production. Modulation of autophagic processes.	[88]
Empagliflozin	Type 2 diabetes mellitus	3 mg/kg orally	Wistar rats	Reduced mechanical, heat, and cold sensitivity, along with improved motor performance. Additionally, axonal damage was minimized, endoneurial edema decreased, inflammatory cell infiltration reduced, and Schwann cells were preserved.	⇑p-AMPK expression ⇓ reducing p-p38 MAPK expression ⇓ p-ERK1/2 ⇓ p-NF-κB p65 ⇓ TNF-α ⇓ IL-1β ⇑ SOD ⇓ MDA ⇓ mTOR ⇑ ULK1 ⇑ beclin-1 ⇓ miR-21 ⇑ RECK ⇓ MMP-2	[89]
Semaglutide	Type 2 diabetes mellitus	1.44 and 2.88 mg/kg orally	Wistar rats	Significantly reduced both mechanical allodynia and thermal hyperalgesia.	⇓ activation of microglia and astrocytes in the dorsal horn ⇓ HbA1c ⇓ AGE ⇓ IL-1β ⇓ IL-6 ⇓ TNF-α	[90]
Atorvastatin	Several types of dyslipidemias; To reduce cardiovascular events in patients with risk factors and/or dyslipidemia.	10, 20, and 40 mg/kg orally	Wistar rats	Higher dose significantly decreased the thermal sensitivity.	Modulation of nitrergic system. ⇓ NO ⇑ MNCV	[91,92]
Alirocumab	Adults with primary hyperlipidemia and homozygous familial hypercholesterolemia and pediatric patients aged 8 years and older with HeFH; To reduce cardiovascular events in patients with risk factors.	10 mg/kg i.p.	Sprague–Dawley rats	Increased intraepidermal nerve fiber density.	Upregulation of the expression of myelin-related genes in sciatic nerve. ⇓ MDA ⇑ SOD ⇓ TNF-α ⇓ IL-1β ⇓ IL-6 ⇑ Sirt1 levels ⇑ NCV ⇓ TG ⇓ CHO ⇓ LDL ⇑ HDL	[93,94]
Drug combinations						
Metformin OR Merformin + duloxetine/ oxycodone/ eslicarbazepine acetate OR Metformin + vitamin B12	Metformin: type 2 diabetes mellitus Duloxetine: - Major depressive disorder; - Generalized anxiety disorder; - Diabetic peripheral neuropathy; - Fibromyalgia; - Relief of chronic musculoskeletal conditions; - Stress urinary incontinence in adult women. Vitamin B12: B12 deficiency due to various causes: - Pernicious anemia; - Celiac disease, gastrectomy; - Small bowel bacterial overgrowth; - Fish tapeworm infection; - Pancreatic or intestinal cancer.	75–250 mg/kg/day orally OR 10–30 μg Intrathecal OR 10–30 μg Intra-plantarly 0.0625–0.5 mg/kg (orally) + 0.0625–0.5 mg/kg (orally) 0.0625–0.5 mg/kg (orally) + 0.15 mg/kg (i.p.)	C57BL/6 mice	Metformin produced dose-dependent antihyperalgic effects in diabetic mice. Combinations dose-dependently reduced mechanical hyperalgesia in a synergistic way. Metformin co-treatment increased analgesic efficacy and enabled the use of lower doses.	Spinal and peripheral mechanism.	[95,96,97]
Aliskiren or Gliclazide or Aliskiren + Gliclazide	Aliskiren: hypertension in adults and pediatric patients aged ≥6 years Glicazide: type 2 diabetes mellitus	45 mg/kg or 25 mg/kg or 45 mg/kg + 25 mg/kg orally	Sprague–Dawley rats	Aliskiren alone and in combination effectively reduced thermal and mechanical hyperalgesia. Gliclazide alone did not produce a significant reduction in DN symptoms.	All the treatments: ⇓ glucose levels ⇓ TSOD ⇓ Mn-SOD ⇓ CAT ⇓ MDA ⇓ AR activities ⇑ GSH level	[98,99,100]
Pioglitazone or Ranolazine	Pioglitazone: type 2 diabetes mellitus Ranolazine: chronic angina	10 mg/kg or 20, 50, and 100 mg/kg orally	Wistar rats	Decreased thermal hyperalgesia and mechanical allodynia, with ranolazine at a dose of 100 mg/kg demonstrating the most advantageous effects.	All treatments: ⇓ TNF-α ⇓ 1L-1B ⇓ levels of Nav 1.7 channels ⇑ spinal PPAR-γ gene expression	[101]

AGE—advanced glycation end-products, AMPK—5′ AMP-activated protein kinase, AR—aldose reductase, CAT—catalase, CHO—cholesterol, GSH—reduced glutathione, HbA1c—glycated hemoglobin, HDL—high-density lipoprotein, HeFH—heterozygous familial hypercholesterolemia, i.p.—intraperitoneal, IL-1β—interleukin-1 beta, IL-6—interleukin-6, LDL—low-density lipoprotein, MDA—malondialdehyde, miRNA—microRNA, MMP-2—matrix metalloproteinase-2, Mn-SOD—manganese-dependent superoxide dismutase, MNCV—motor nerve conduction velocity, mTOR—mammalian target of rapamycin, NCV—nerve conduction velocity, NF-κB—nuclear factor kappa B, NO—nitric oxide, p-AMPK—phosphorylated AMP-activated protein kinase, p-ERK1/2—phosphorylated extracellular signal-regulated kinase 1/2, p-NF-κB p65—phosphorylated NF-κB p65 subunit, p-p38 MAPK—phosphorylated p38 mitogen-activated protein kinase, PPAR-γ—peroxisome proliferator-activated receptor gamma, RECK—reversion-inducing cysteine-rich protein with Kazal motifs, RSCs—rat Schwann cells, ROS—reactive oxygen species, Sirt1—sirtuin 1, SC—spinal cord, SNCV—sensory nerve conduction velocity, SOD—superoxide dismutase, TG—triglycerides, TNF-α—tumor necrosis factor-alpha, TSOD—total superoxide dismutase, ULK1—Unc-51-like autophagy activating kinase 1.

**Table 2:** Anti-oxidative and mitochondrial protective agents with overlapping anti-inflammatory and neuroprotective actions in animal models of DN.

Tested Substance	Approved Indication	Mode of Administration	Animal Strain	Results	Molecular Mechanism	Reference
Liraglutide	Type 2 diabetes mellitus	2 μg/2 μL into the right lateral ventricle	GSK3β(S9A) mice	Significantly ⇓ mechanical allodynia and thermal nociception in WT mice, while the analgesic effects were absent in GSK3β(S9A) mice.	GSK3β activation. Subsequent inhibition of NLRP3 inflammasome in brain microglia.	[102]
		0.4 mg/kg i.p.	ICR mice	Alleviated antinociceptive behaviors in the second phase of formalin test. Decreased mechanical sensitivity.	⇓ neopterin levels ⇑ glutamine/glutamate ratio	[103]
Topiroxostat	Gout and hyperurcemia	1 and 2 mg/kg orally	db/db mice	Preserved nerve conduction, intraepidermal nerve fiber density, and neurite outgrowth. Reduced inflammation and oxidative stress in a dose-dependent manner	Suppressed proinflammatory M1 macrophage polarization. ⇓ mRNA expression of TNF-α, IL-1β, and iNOS. ⇓ H_2_O_2_ and MDA levels in plasma and sciatic nerve. ⇓ 8-OHdG expression in Schwann cells, endothelial cells, and macrophages.	[104,105]
Amlodipine besylate	- Hypertension; - Coronary artery disease; - Chronic stable angina; - Vasospastic angina.	2 and 4 mg/kg/day orally	C57BL/6N mice	Ameliorated 50% paw withdrawal threshold, thermal response latency, motor nerve conduction, and sensory nerve conduction velocity Improved intraepidermal nerve fiber loss and myelin sheath structural damage.	GPR40 activation. Suppressed neuroinflammation via the GPR40/β-arrestin2/NLRP3 pathway. Ameliorated mitochondrial dysfunction through the GPR40/LKB1/AMPK/SIRT1/PGC-1α pathway.	[106,107]
Rilmenidine	Hypertension	0.1 and 0.2 mg/kg orally	Sprague–Dawley rats	Decreased perineural thickness and improved muscle activity as evidenced by EMG analysis in a dose-dependent manner	⇓ TNF-α ⇓ HMGB-1 ⇓ MDA ⇑ NGF ⇑ LC-3	[17]
Molsidomine	- Angina; - Ischemic heart disease; - Chronic heart failure; - Pulmonary hypertension;	5 and 10 mg/kg orally	Wistar rats	Improved motor coordination, mechanical allodynia, and hyperalgesia.	Prevention of reduced GSH depletion in the sciatic nerve. ⇓ HbA1c ⇓ MDA ⇑ NCV	[108,109]
Trimetazidine	Stable angina	10 and 20 mg/kg i.p.	Sprague–Dawley rats	Significant increase in CMAP amplitudes and a markedly shorter CMAP latency	⇓ HMGB1 ⇓ pentraxin-3 ⇓ TGF-ß ⇓ MDA	[110,111]
Vincamine	Management and prevention of cerebrovascular diseases	30 mg/kg i.p.	C57BL/6J mice	Improved neurological dysfunctions (as assayed against tactile allodynia, thermal response test, MNCV, and peripheral vascular dysfunctions). Ameliorated myelin sheath injury of sciatic nerve tissues.	Activated GPR40. Alleviated inflammation involving GPR40/β-arrestin2/NLRP3 and GPR40/β-arrestin2/IκBα/NF-κB/NLRP3 pathways. Improved mitochondrial respiration impairments in dorsal root ganglion neurons and mitochondrial dysfunction through GPR40/CaMKKβ/AMPK/SIRT1/PGC-1α pathway and oxidative stress through GPR40/Nrf2 pathway.	[112]
Melatonin	- Sleep disturbances - Circadian rhythm disruptions - Supportive role in benzodiazepine withdrawal syndrome	10 mg/kg orally	KM mice	Significantly alleviated neuronal death and inhibited neuronal pyroptosis and excessive autophagy.	Modulated miR-214-3p/caspase-1 and miR-214-3p/ATG12 pathways. ⇓ levels of NLRP3 ⇓ cleaved caspase-1 ⇓ GSDMD-N ⇓ IL-1β ⇓ LC3, beclin1, and ATG12.	[113,114]
Tropisetron	Prevention of chemotherapy-induced and postoperative nausea and vomiting.	3 mg/kg i.p.	Wistar rats	Reversed the thinning of nerve fibers and myelin sheaths and preserved the number of myelinated fibers.	⇓ TNF-α ⇓ IL-1β ⇑ Bcl-2 expression ⇓ Bax expression.	[115,116]
Topiramate	- Partial-onset or generalized tonic–clonic seizures - Adjunctive treatment for Lennox–Gastaut syndrome; - Migraine prophylaxis	10–30 mg/kg orally	Swiss mice	Increased hotplate latency time and von Frey test pain threshold.	⇓ neuroinflammation, by inhibiting the activation of glial cells in the spinal cord. ⇑ the expression of GAP-43 and neurofilaments.	[18,117]
Romidepsin	Cutaneous T-cell lymphoma	1 mg/kg i.p.	ob/ob mice	Alleviated thermal hyperalgesia and mechanical allodynia.	Inhibited histone deacetylase. Modulated immune-related markers such as neutrophil elastase, cfDNA, CitH3, and PADI4. Modulated the expression of GAP-43 and GLUT-4, promoting nerve regeneration.	[118,119]

8-OHdG—8-hydroxy-2′-deoxyguanosine, ATG12—autophagy-related gene 12, Bax—Bcl-2-associated X protein, Bcl-2—B-cell lymphoma 2, CaMKKβ—calcium/calmodulin-dependent protein kinase kinase β, cfDNA—cell-free DNA, CHO—cholesterol, CitH3—citrullinated histone H3, CMAP—compound muscle action potential, db/db—leptin receptor-deficient diabetic mouse model, EMG—electromyography, GAP-43—growth-associated protein 43, GLUT-4—glucose transporter type 4, GSDMD-N—gasdermin D N-terminal, GSK3β—glycogen synthase kinase-3 beta, GSK3β(S9A)—constitutively active mutant of glycogen synthase kinase-3 beta (serine 9 to alanine), HbA1c—glycated hemoglobin, HMGB1—high mobility group box 1, H_2_O_2_—hydrogen peroxide, ICR—Institute of Cancer Research mouse strain, IL-1β—interleukin-1 beta, iNOS—inducible nitric oxide synthase, i.p.—intraperitoneal, IκBα—inhibitor of kappa B alpha, LC-3—microtubule-associated protein 1A/1B-light chain 3, MDA—malondialdehyde, miRNA—microRNA, MNCV—motor nerve conduction velocity, NCV—nerve conduction velocity, NGF—nerve growth factor, NLRP3—NOD-like receptor family pyrin domain containing 3, NO—nitric oxide, Nrf2—nuclear factor erythroid 2–related factor 2, PADI4—peptidylarginine deiminase 4, PGC-1α—peroxisome proliferator-activated receptor gamma coactivator 1-alpha, SIRT1—sirtuin 1, TGF-ß—transforming growth factor beta, TNF-α—tumor necrosis factor-alpha, WT—wild type.

**Table 3:** Agents targeting neuroinflammation, cytokine signaling, and central sensitization in animal models of DN.

Tested Substance	Approved Indication	Mode of Administration	Animal Strain	Results	Molecular Mechanism	Reference
Pentoxifylline	Intermittent claudication in peripheral arterial disease	50, 100, and 200 mg/kg in drinking water	Wistar rats	Attenuated tactile allodynia dose-dependently. Prevented the reduction in epidermal thickness of footpad skin.	⇓ TNFα ⇓ NF-κB ⇓ microglial Iba1 immunoreactivity	[120,121]
Pramipexole	Parkinson’s disease	0.25 and 1 mg/kg orally	Sprague–Dawley rats	Improved survival rate and effectively restored normal structural characteristics of sciatic tissue in a dose-dependent manner.	⇑ NGF ⇓ MDA ⇓ NO ⇑ reduced GSH levels ⇓ sciatic nerve tissue expression of TLR4/MyD88/IRAK-1/TRAF-6/NF-κB axis	[122]
Telmisartan	Hypertension	5 and 10 mg/kg orally	Wistar rats	Reduced mechanical nociceptive threshold, motor coordination, and thermal nociceptive threshold. Prevented nerve degeneration.	⇑ NGF ⇓ TNF-α ⇓ IL-1β ⇓ IL-6	[123]
Clavulanic acid	Enhancement of antibiotic antibacterial efficacy.	10, 20, and 40 mg/kg i.p.	Wistar rats	Reduced mechanical and cold allodynia, as well as thermal hyperalgesia, in both prophylactic and therapeutic regimens across all doses.	⇑ GLT1 ⇓ iNOS ⇓ TNF-α ⇓ Bax/Bcl2 ratio	[124,125]
Reboxetine	Clinical depression	8 and 16 mg/kg orally	Sprague–Dawley rats	Alleviated mechanical and thermal allodynia and thermal hyperalgesia.	Activation of β_2_-adrenoceptors. Modulation of D_1_, D_2_, and D_3_ dopaminergic and δ-opioid receptors. ⇑ noradrenaline levels	[126]
Ifenprodil	Neuropsychiatric conditions, notably dependence and depression	0.5 and 1.0 μg intrathecally	Sprague–Dawley rats	Formalin-induced flinching and licking behavior significantly decreased in a dose-dependent manner.	Inhibition of microglia activation. Downregulation of DREAM protein expression. Reduction of BDNF expression.	[127,128]
Etifoxine	- Anxiety disorders - Enhancement of peripheral nerve repair	50 mg/kg i.p.	C57BL6/J mice	Reduced STZ-induced mechanical allodynia. Analgesic effect persisted five weeks after the end of the treatment.	⇓ in pro-inflammatory cytokine production, microglial activation, or neuroprotective properties.	[129,130]
Bupivacaine	Local or regional anesthesia	0.01%, 0.1%, and 1%/400 μL through the dorsocaudal region of the spine.	C57BL/6 J mice	Ameliorated mechanical allodynia, thermal hyperalgesia, and thermal allodynia, demonstrating greater effectiveness at lower concentrations.	Regulation of miR-23a/PDE4B axis. ⇓ TNFα, IL-6, IL-1β, and MCP-1 (greater effect at lower concentration).	[131,132]
Drug combinations						
Rolipram + Pentoxifylline	Rolipram: clinical depression Pentoxifylline: intermittent claudication in peripheral arterial disease	1 mg/kg or 100 mg/kg or 0.5 mg/kg + 50 mg/kg	Sprague–Dawley rats	Monotherapy and association significantly attenuated motor function deficiency by modulating distance moved and velocity. They increased cAMP levels. The combination treatment demonstrated the maximum effectiveness.	All treatments: ⇓ LPO ⇓ ROS ⇓ TNF-α ⇓ NF-kB ⇓ COX2 ⇓ TAC ⇑ total thiol ⇑ CAT ⇑ SOD	[121,133,134]
Sildenafil + Metformin	Sildenafil: -Erectile dysfunction; -Pulmonary hypertension; Metformin: type 2 diabetes mellitus	2, 2.5, and 3 mg/kg + 100, 300, and 500 mg/kg orally	Wistar rats	All combinations resulted in reduced thermal and tactile sensitivity.	⇓ TNF-α ⇓ IL-6 levels ⇓ nitrite levels ⇑ total thiols concentration	[135,136]
		2, 2.5, and 3 mg/kg + 100, 300, and 500 mg/kg orally	NMRI mice	All combinations significantly ⇑ pain reaction latencies in the hot-plate and tail withdrawal tests in a dose-dependent manner.	⇓ glucose levels. ⇓ nitrite levels in brain and liver.	[136,137]

Bax—Bcl-2-associated X protein, Bcl-2—B-cell lymphoma 2, BDNF—brain-derived neurotrophic factor, CaMKKβ—calcium/calmodulin-dependent protein kinase kinase β, cAMP—cyclic adenosine monophosphate, CAT—catalase, CHO—cholesterol, COX2—cyclooxygenase-2, DREAM—downstream regulatory element antagonist modulator protein, D_1_—dopamine receptor D1, D_2_—dopamine receptor D2, D_3_—dopamine receptor D3, δ—delta opioid receptor, GAP-43—growth-associated protein 43, GLT1—glutamate transporter 1, GSH—reduced glutathione, IL-1β—interleukin-1 beta, IL-6—interleukin-6, iNOS—inducible nitric oxide synthase, i.p.—intraperitoneal, IRAK-1—interleukin-1 receptor-associated kinase 1, Iba1—ionized calcium-binding adapter molecule 1, MCP-1—monocyte chemoattractant protein-1, MDA—malondialdehyde, miR-23a—microRNA-23a, miRNA—microRNA, NGF—nerve growth factor, NF-κB—nuclear factor kappa B, NO—nitric oxide, ROS—reactive oxygen species, SOD—superoxide dismutase, TAC—total antioxidant capacity, TGF-β—transforming growth factor beta, TLR4—toll-like receptor 4, TNF-α—tumor necrosis factor-alpha, TRAF-6—TNF receptor-associated factor 6.

**Table 4:** Ion channel modulators and regulators of neuronal hyperexcitability in animal models of DN.

Tested Substance	Approved Indication	Mode of Administration	Animal Strain	Results	Molecular Mechanism	Reference
Memantine	Alzheimer’s disease	10 mg/kg orally	Swiss albino mice	Alleviated thermal hyperalgesia and mechanical allodynia.	Inhibited excessive activation of NMDAR1 receptors. ⇓ HMGB1/TLR4/NF-kB inflammatory pathway activity. ⇓ glutamate levels. ⇓ TNF-α and IL-1β in the spinal cord.	[138,139]
Retigabine	Adjunct therapy for partial-onset seizures	15 mg/kg i.p.	Sprague–Dawley rats	Significantly attenuated mechanical, not heat, hypersensitivity.	Activation of Kv7 channels	[140,141]
Cilostazol	Intermittent claudication	10, 30, and 100 mg/kg orally	Sprague–Dawley rats	Alleviated mechanical allodynia symptoms but had no effect on thermal analgesia.	30 and 100 mg/kg: ⇓ PGP9.5 epidermal sensory nerve fiber disruptions in the hind paws. ⇓ P2X3, CGRP, and TRPV-1 expression in the hind paws. 100 mg/kg: Amelioration of microglial overreaction and restoration of the reduced astrocyte expression.	[142,143]
N-Acetylcysteine	- Mucolysis; - Paracetamol intoxication.	50 and 100 mg/kg i.p.	C57BL/6 mice	Reduces mechanical nociceptive threshold at the dose of 100 mg/kg.	TRPV1, NMDAR, and mGlu5 antagonism. ⇓ RK1/2 phosphorylation ⇓ P2X7 receptor expression in the spinal cord	[144,145]
Pirenzepine	Peptic/gastric/ duodenal ulcer	0.2, 1.0, 2.0, or 10% in 50 μL hydrogel locally (hind paw)	C57 BL/6J mice	2.0%: Prevented paw tactile allodynia, heat hypoalgesia, and intraepidermal nerve fiber loss. 10%: Prevented paw tactile allodynia, heat hypoalgesia, and IENF loss.	Muscarinic M1 receptor antagonism	[146,147]
Oxybutynin	Overactive bladder	3–10 mg/kg/day s.c./ 3% gel topical	Swiss Webster mice	Reversed paw heat hypoalgesia. Prevented nerve loss.	Muscarinic M1 receptor antagonism	[148,149]
Atropine	Systemic administration: muscarinic intoxication. Ophthalmic use: -Mydriasis; -Cycloplegia.	2.0% in 50 μL hydrogel topically	Swiss Webster mice	Paw delivery: prevented MNCV slowing, heat hypoalgesia, and loss of IENF in the ipsilateral limb, but not in the contralateral. Ocular delivery: prevented or attenuated paw heat hypoalgesia in both hind paws, but not MNCV slowing, tactile allodynia, or loss of IENF.	Muscarinic M1 receptor antagonism	[146,150]
Drofenine	Visceral spastic pain	10, 20 mg/kg i.p.	C57BL/6 mice	Attenuated nerve conduction deficiency, hypoalgesia, impairment in MNCV, enhanced sensitivity to mechanical stimuli, and augmented thermal response.	Kv2.1 inhibition	[151]
		10, 20 mg/kg i.p.	db/db mice	Attenuated nerve conduction deficiency, hypoalgesia, impairment in motor nerve conduction velocity, enhanced sensitivity to mechanical stimuli, and augmented thermal response.	⇓ Kv2.1 expression, promoting neurite outgrowth. ⇓ IκBα/NF-κB signaling ⇓ apoptosis by regulating Bcl-2 and caspase-3 expression. Modulation of Kv2.1/CaMKKβ/AMPK/PGC1α pathway.	[151]
Pioglitazone	Type 2 diabetes mellitus	100 mg/kg i.p., single dose or 0.3, 3, or 30 mg/kg/day in chow	db/db mice	Decreased heat hypersensitivity in db/db mice with greater efficacy in female mice after single i.p. dose. Neither 0.3, 3.0, nor 30 mg/kg/day decreased heat hypersensitivity in males or females.	PPARγ activation	[152]
Drug combinations						
Agomelatine or Morphine or Agomelatine + Morphine	Agomelatine: major depressive episodes Morphine: chronic pain ranging from moderate to severe	10 mg/kg, each i.p.	Balb/C mice	All the treatments attenuated thermal sensitivity. The combination produced a stronger and longer-lasting analgesic effect compared to morphine alone. Repeated exposure to morphine leads to a reduced analgesic effect, requiring higher doses to achieve the same level of pain relief.	Agomelatine: ⇓ GluN1 expression in the PAG and dorsal raphe, contributing to ⇓ morphine tolerance.	[153,154,155]
Melatonin or Melatonin + Gabapentin	Melatonin: - Sleep disturbances - Circadian rhythm disruptions, - Withdrawal symptoms from benzodiazepines and nicotine. Gabapentin: - Partial-onset seizures, - Peripheral neuropathic pain.	25 and 50 mg/kg or 50 mg/kg + 100 mg/kg orally	Sprague–Dawley rats	The combination treatment significantly relieved cold allodynia, while both melatonin alone and the combination effectively alleviated hot allodynia.	Melatonin 25 mg/kg: ⇑ hepatic gene expression of PGC-1α and TFAM ⇑ SNCV Melatonin 50 mg/kg: ⇑ hepatic gene expression of PGC-1α and TFAM ⇑ SNCV ⇓ serum creatinine levels The combination: ⇑ SNCV	[113,156,157]

CGRP—calcitonin gene-related peptide; Caspase-3—cysteine–aspartic acid protease 3; GluN1—NMDA receptor subunit 1; HCN2—hyperpolarization-activated cyclic nucleotide-gated channel 2; HMGB1—high mobility group box 1; IENF—intraepidermal nerve fiber; Kv2.1—potassium voltage-gated channel subfamily B member 1; Kv7—potassium voltage-gated channel subfamily KQT member; NMDAR1—N-methyl-D-aspartate receptor subtype 1; P2X3—purinergic receptor P2X, ligand-gated ion channel, 3; P2X7—purinergic receptor P2X, ligand-gated ion channel, 7; PAG—periaqueductal gray; PGP9.5—protein gene product 9.5; PPARγ—peroxisome proliferator-activated receptor gamma; RK1/2—ribosomal S6 kinase 1 and 2; SNCV—sensory nerve conduction velocity; TFAM—mitochondrial transcription factor A; TLR4—toll-like receptor; TRPV-1—transient receptor potential vanilloid 1.1.

## Data Availability

All data generated or analyzed during this study are included in this published article.
